# Reactive Oxygen Species and Pressure Ulcer Formation after Traumatic Injury to Spinal Cord and Brain

**DOI:** 10.3390/antiox10071013

**Published:** 2021-06-24

**Authors:** Suneel Kumar, Thomas Theis, Monica Tschang, Vini Nagaraj, Francois Berthiaume

**Affiliations:** 1 Department of Biomedical Engineering, Rutgers, The State University of New Jersey, Piscataway, NJ 08854, USA; fberthia@soe.rutgers.edu; 2 Keck Center for Collaborative Neuroscience, Department of Cell Biology and Neuroscience, Rutgers University, Piscataway, NJ 08554, USA; theis@dls.rutgers.edu (T.T.); vn149@dls.rutgers.edu (V.N.); 3 School of Art and Sciences, Rutgers, The State University of New Jersey, Piscataway, NJ 08854, USA; mat361@scarletmail.rutgers.edu

**Keywords:** spinal cord injury, brain injury, pressure ulcer, reactive oxygen species, oxidative stress, wound healing

## Abstract

Traumatic injuries to the nervous system, including the brain and spinal cord, lead to neurological dysfunction depending upon the severity of the injury. Due to the loss of motor (immobility) and sensory function (lack of sensation), spinal cord injury (SCI) and brain injury (TBI) patients may be bed-ridden and immobile for a very long-time. These conditions lead to secondary complications such as bladder/bowel dysfunction, the formation of pressure ulcers (PUs), bacterial infections, etc. PUs are chronic wounds that fail to heal or heal very slowly, may require multiple treatment modalities, and pose a risk to develop further complications, such as sepsis and amputation. This review discusses the role of oxidative stress and reactive oxygen species (ROS) in the formation of PUs in patients with TBI and SCI. Decades of research suggest that ROS may be key players in mediating the formation of PUs. ROS levels are increased due to the accumulation of activated macrophages and neutrophils. Excessive ROS production from these cells overwhelms intrinsic antioxidant mechanisms. While short-term and moderate increases in ROS regulate signal transduction of various bioactive molecules; long-term and excessively elevated ROS can cause secondary tissue damage and further debilitating complications. This review discusses the role of ROS in PU development after SCI and TBI. We also review the completed and ongoing clinical trials in the management of PUs after SCI and TBI using different technologies and treatments, including antioxidants.

## 1. Introduction

Pressure ulcers (PUs) are skin injuries primarily caused by prolonged pressure on the skin and underlying tissue. Skin wound healing is a multi-stage process that aims to restore the integrity and function of the skin after injury but is often impaired in patients with underlying diseases or other medical conditions, including traumatic brain injury (TBI) and spinal cord injury (SCI). TBI and SCI result in sensory (lack of sensation) and motor loss (paralysis). Affected patients are at risk of PU development because they do not feel the pain that would normally signal excessive and prolonged pressure in one area and lack mobility to alter position to relieve that pressure. PUs are prone to reoccur and may lead to rehospitalization, which increases the cost of care and burdens the families of patients both financially and emotionally.

In the United States, 1–3 million people per year develop PUs [1]. It causes enormous costs to the healthcare system, with an average of over $124,000 per patient to treat a stage 4 PU [2] and a total annual cost of up to $11 billion [3]. The severity of PUs is scored using different stages (stages 1–4). Stage 1 is the mildest form of PU and, in many cases, it can transform into a severe stage such as stage 4. Stage 4 is the most severe form of PU with full-thickness skin loss and extensive destruction of the tissue that affects also an underlying muscle, bone, and/or supporting tissue [4]. From nursing home admissions, 10–13% have PU ratings if in stages 2 to 4 [5]. PUs are caused by immobility and lack of protective sensory perception after any neurological injury; 28.3% of patients that suffer from SCI and 18.8% of patients with TBI develop PUs during their hospitalization [3,6]. In the lifetime of SCI patients, up to 95% may develop advanced stages 3 or 4 PUs [7]. Patients with a cervical or thoracic SCI have a three-fold greater risk to develop a PU compared to patients with a lumbar or sacral SCI [8]. The severity of TBI can be scored with the GCS scale [9]. The percentage of TBI patients with PU development varies from mild to severe TBI. As mild TBI patients are lower in the number who develop PU compared to moderate and severe TBI patients. Severe TBI patients with PU had a five times higher mortality [10], as compared to mild and moderate TBI.

In this review, we discuss the latest updates on the role of traumatic injury in the development of PUs, and the underlying mechanisms involving reactive oxygen species (ROS) and oxidative stress. We also discuss the results of ongoing clinical trials for the treatment of PU after SCI and TBI.

## 2. Spinal Cord Injury and Pressure Ulcers

One of the major reasons for the rehospitalization of SCI patients who are over 25 years old is due to the development of PUs [11]. The European Pressure Ulcer Advisory Panel, the Pan Pacific Pressure Injury Alliance, and the National Pressure Ulcer Advisory Panel jointly published international clinical practice guidelines to prevent and treat PUs [12]. Coleman and colleagues suggest that there are three direct causal factors of developing a PU: (1) Immobility; (2) general health condition of the skin and if the patient has a history of developing PUs; (3) how well the tissue is perfused. They also identified several indirect causal factors, including moisture, sensory perception, diabetes, age, low albumin, and poor nutrition [13].

### 2.1. Underlying Mechanisms in the Development of Pressure Ulcers

The primary cause of developing a PU is a mechanical load on the skin over bones causing localized pressure and shear [13]. These mechanical forces trigger different damage mechanisms that cause damage to the tissue [14]. Mechanical deformation of the tissue may induce immediate damage to the cells. The magnitude of the deformation is the most important factor that determines the severity of the damage [15,16,17]. Prolonged loading periods lead to occlusion of blood vessels, causing ischemia, a reduced supply of nutrients, and the accumulation of metabolites, all of which leading to tissue damage [15,18,19,20]. Changes in the body position of a patient remove the mechanical force on the skin, which leads to reperfusion of the tissue. This reperfusion might exacerbate the damage caused by ischemia through the release of harmful ROS [21,22]. The occlusion of lymph vessels in soft tissues also contributes to the accumulation of waste products and the buildup of interstitial fluid which further promotes the development of a PU [23,24]. Two major routes for PU development have been described. First, the pathway of a superficial PU starts with superficial loss of the epidermis caused by shear stresses on the skin layers. If the mechanical force remains on the skin, the damage will progress to deeper tissues. The second pathway starts with deep tissue injury with necrosis of muscle and fat that is triggered by mechanical deformation and sustained compression of soft tissues. This deep damage eventually leads to the destruction of the superficial layers, at which point the full depth of the ulcer is visible [14,25].

### 2.2. Role of Oxidative Stress in the Development of Pressure Ulcers

Ischemia-reperfusion leads to a transient ROS spike in the tissue. ROS are products of cellular metabolism that contain radical and non-radical derivatives of oxygen. Oxygen radicals contain one or more unpaired electrons and thus, are the most unstable and highly reactive; these include superoxide, hydroxyl, peroxyl, and hydroperoxyl. Non-radicals include hydrogen peroxide and peroxynitrite. An increase of ROS in the cell might lead to inappropriate oxidation of DNA, proteins, and lipids, which might lead to loss of function and potentially to an additional increase of ROS levels. To regulate ROS levels in the cell, antioxidant mechanisms have evolved. Excessive ROS levels and ensuing oxidative stress arise if the production of ROS increases and/or when antioxidants are depleted [26,27].

The free radical superoxide occurs if an additional electron is added to the outer orbital of the oxygen atom. This may happen in the mitochondrial electron transport chain or during the NADPH dehydrogenase reaction. Under normal physiological conditions, there occurs leakage of 1–3% superoxide from the mitochondria into the cytoplasm. This leakage increases with higher oxygen levels in the cell and is dependent upon the cellular rates of energy substrate and energy utilization [28]. Superoxide can also be produced by enzymes such as NADPH oxidase and xanthine oxidase. NADPH oxidase is expressed in phagocytic cells, such as neutrophils, macrophages, and sensitized monocytes activated by inflammatory stimuli. Phagocytic cells purposely secrete hydrogen peroxide to fight pathogens but this may also cause local tissue damage [29]. Non-phagocytic cells, such as fibroblasts, smooth muscle cells, endothelial cells, and chondrocytes also express NADPH oxidase which is a component of their intracellular signal cascades [30,31,32,33,34]. An increase in NADPH oxidase in the tissue will cause more free radicals to be generated [35].

The non-radical H_2_O_2_ is produced by peroxisomes, and during high peroxisome activity, hydrogen peroxide can leak into the cytoplasm [36]. H_2_O_2_ is also formed upon dismutation and simultaneous oxidation and reduction of superoxide. Hydrogen peroxide can be also produced by xanthine oxidase, urate oxidase, and D-amino acid oxidase. Although hydrogen peroxide is relatively unreactive, it has a long half-life time (cellular half-life ~1 ms, steady-state levels ~10^−7^ M) [37], it can cross cellular membranes and can react with transition metals such as iron to form the highly reactive hydroxyl radical according to the Haber-Weiss reaction [38]. The reaction with ferrous ions is called the Fenton reaction. Since hydrogen peroxide can cross the plasma membrane, hydrogen peroxide and secondarily derived hydroxyl radicals can affect neighboring cells. Copper is another transition metal that forms complexes with proteins, DNA, and carbohydrates. In a complex, copper can undergo redox cycling and can function as an active site to form hydroxyl radicals [39]. This can have different consequences depending on which cell compartment and what kind of copper-macromolecules are affected. In the plasma membrane, it can cause a cascade of lipid radical formation and the oxidation of lipids would lead to damage of the lipid bilayer. Oxidized proteins get rapidly digested in peroxisomes by the proteolytic system. Overactivation of this system leads to increased leakage of hydrogen peroxide from the peroxisomes, more cellular damage, and might lead to apoptosis [40]. Copper-DNA complexes can lead to DNA damage [41].

The nitrogen-centered free radical nitric oxide, which is produced by the enzyme nitric oxide synthase, has an ambivalent role [42]. It can act both as an oxidant and as an antioxidant. It. As an oxidant, it can form reactive nitrogen species such as peroxynitrite, nitrosonium cation, and nitroxyl anion [43]. An extensive generation of these nitric oxide-related radicals can relate to cell injury. For example, peroxynitrite can oxidize lipids that affect cell membranes, aromatic amino acids in proteins that affect their structures, and sulfur-containing amino acids which lead to a depletion of the antioxidant glutathione [44]. At low concentrations, nitric oxide acts as an antioxidant. By reacting with and neutralizing oxidants, nitric oxide attenuates the Fenton reaction. A high concentration of nitric oxide would be able to scavenge the oxidant peroxynitrite, which would result in dinitrogen trioxide. This would lead to the attenuation of the pH-independent reaction of peroxynitrite and carbon dioxide to the oxidant CO_2_OONO_2_ [45]. Xanthine oxidase is the enzyme that catalyzes the oxidation of hypoxanthine to xanthine and the oxidation of xanthine to uric acid [46]. Although under normal conditions it is not a major contributor to ROS generation, there are indications that it is an important ROS generator during ischemia-reperfusion [47,48,49].

The epidermis of the skin provides major protection from oxidative damage. Hydrophilic, lipophilic, and enzymatic antioxidants are present at higher concentrations in the epidermis than in the dermis layer of the skin [50]. In the epidermis, there is a concentration gradient of antioxidants [51,52]. The uppermost tissue layer, which is exposed to the environment, has the lowest concentration of antioxidants, and this concentration increases with the increasing depth of the epidermis. Additionally, antioxidant levels can be enhanced by exogenous antioxidant treatments, such as vitamin C acetate and/or vitamin E [51,52].

**Figure 1:**
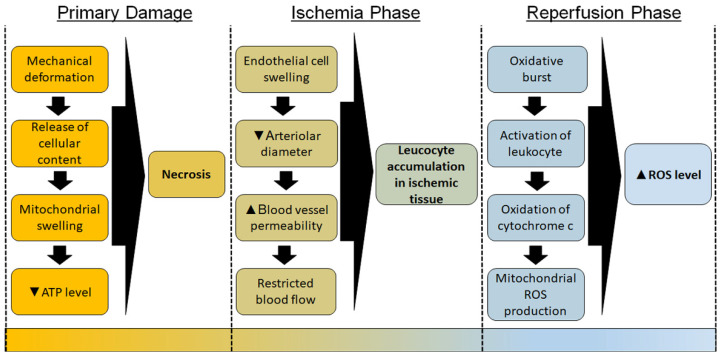
Timeline of the pathology in the development of chronic pressure ulcer (PU). The scheme displays the chronological order of pathological events that occur during the primary damage of the skin, the ischemia phase, and the reperfusion phase that lead to the development of PU. ▲—Increase; ▼—Decrease.

Primary damage to the skin occurs after severe mechanical deformation of the tissue. Plasma membranes rupture, which leads to a release of cellular content into the extracellular space, cellular and mitochondrial swelling, ATP levels drop, thus leading to necrosis of the tissue [53]. Subsequently, ischemia-reperfusion of the skin induces additional damage. During the ischemic phase, endothelial cells swell, which leads to a reduction of the arteriolar diameter, restricted blood flow, and/or increased permeability of the blood vessels [54] As a consequence, leucocyte adhesion increases in ischemic tissue, causing neutrophils to attach to the walls of the blood vessels or to be trapped in the capillary bed [55,56]. Major players in this ROS production are xanthine oxidase and phagocytic cells, mainly neutrophils. During ischemia, xanthine dehydrogenase is converted to xanthine oxidase. During the reperfusion phase, oxygen is reintroduced to the tissue, which leads to an oxidative burst that produces an extensive amount of ROS [49]. After oxygen returns to the tissue, xanthine oxidase generates superoxide and hydrogen peroxide, which causes tissue injury, activation of phagocytic cells, and damage to membrane lipids, proteins, and DNA [57]. In addition, the return of oxygen into the tissue leads to a dramatic increase in mitochondrial activity. Release of cytochrome c from mitochondria to cytoplasm leads to a massive ROS production by the mitochondrial respiratory chain during the reperfusion phase [58]. Nitric oxide reacts with superoxide to form peroxynitrite [59,60]. ROS can trigger neutrophil infiltration and activation which leads to additional ROS secretion from immune cells [61,62]. The type 2 isoform of nitric oxide synthase (iNOS) is expressed by immune cells and it can be activated by inflammatory stimuli such as cytokines, lipopolysaccharides, and immunological factors [63]. Neutrophils and macrophages also express myeloperoxidase, which is triggered by inflammatory stimuli, leads to the production of the extremely damaging hypochlorous acid [64]. The damage caused by reperfusion injury can be attenuated if neutrophils are depleted from the blood or are otherwise inhibited [65,66]. All these related events are summarized in a chronicle order in Figure 1.

Antioxidants are how cells protect against oxidative stress and buffer the increased ROS levels. Antioxidants can act by enzymatic removal of free radicals, by transition metal ion chelation, or by simply sacrificing themselves by reacting with the free radicals to neutralize them [67]. Superoxide cannot cross the plasma membrane, which can lead to its accumulation inside the cell. The enzyme superoxide dismutase accelerates the dismutation of superoxide to hydrogen peroxide, which can cross the plasma membrane and leak out of the cell. Thus, these processes lead to the removal of the superoxide radical from the site of production [68]. Superoxide dismutase itself is vulnerable to oxidative inactivation, with an ensuing increase in ROS levels in the mitochondria, leading to mitochondrial dysfunction and death [69]. Extracellular superoxide dismutase has an important role in protecting the extracellular matrix against oxidation by superoxide that is secreted from immune cells [70]. Another example of the enzymatic removal of ROS is by the enzyme catalase. This heme-containing enzyme is mainly present in peroxisomes and cleaves hydrogen peroxide to water and oxygen [71].

Glutathione is a small molecule antioxidant, and it protects the thiol groups in active sites of many enzymes [72]. Glutathione peroxidase is an enzyme that breaks down hydrogen peroxide to water and oxygen in the cytoplasm and mitochondria and can also neutralize lipid peroxides by using reduced glutathione. The resulting oxidized glutathione can be regenerated back to reduced glutathione by glutathione reductase [73]. Reduced glutathione can also act as a substrate for glutathione S-transferase, which removes oxidized substrates by reacting them with glutathione molecules. Ferric ion binding proteins such as transferrin and lactoferrin can function as antioxidants by depleting the free ion in the cell and thus inhibiting the Fenton reaction [74]. Albumin, which is present at high concentrations in the blood, has one thiol group that is capable of binding to ROS thus enabling it to function as a scavenger of free radicals [35]. Various small chemical compounds bind to oxidants and thus function as antioxidants. These sacrificial antioxidants can be derived from the diet or are endogenously expressed in the cells. Vitamin C, E, and carotenoids are examples of diet-derived antioxidants. Uric acid and bilirubin are endogenously expressed antioxidants [75,76].

Apoptosis is the programmed cell death that can be triggered by ROS. The caspase family of proteases, and transcription factors such as NF-kappa B, MAPK, and p51, are important signaling molecules for apoptosis. Antioxidants inhibit signaling cascades that lead to apoptosis [77,78]. Neutrophils undergo apoptosis after the oxidative burst which might be triggered by the increased ROS levels [79,80]. Apoptosis can also be initiated by inflammatory signals, UV-light, or specific drugs [81,82]. Reduced glutathione removes hydrogen peroxide in the cytoplasm, and its depletion is associated with the initiation of apoptosis [83,84]. At lower oxidant concentrations, signal pathways will be a trigger that leads to apoptosis. However, if oxidant concentration increases, signal transducer molecules of the apoptosis pathways will also be oxidized and damaged so that the cell will not undergo apoptosis. Instead, the cell will undergo necrosis after the cellular ATP levels are depleted and oxidation caused lethal damage [85,86,87,88].

### 2.3. Impaired Healing of Pressure Ulcers

In patients with SCI, major risk factors to developing a chronic PU with impaired healing are the immobility of the patient together with impaired sensation as well as the general condition of the skin. The risk is exacerbated by increasing age and by metabolic disorders such as diabetes. Following the primary mechanical injury, excessive humidity, immunodeficiency, and diabetes may result in an increased likelihood of bacterial infection of the skin. Such infections trigger an immune response that further increases ROS levels due to secretion from host phagocytes with ensuing additional tissue damage [89,90,91].

Skin aging, which alters the composition of antioxidants, has two types: photoaging, and chronological aging [92]. However, it is more likely that PUs happen in chronologically aged skin compared to photoaged skin. Chronological aging affects skin the same way that it affects other tissues. Enzymatic antioxidants tend to increase, while non-enzymatic antioxidants tend to decrease with aging [93]. During chronological aging, glutathione reductase increases in the epidermis, catalase increases in the epidermis and decreases in the dermis, α-tocopherol decreases in the epidermis, and ascorbate and glutathione decrease in the epidermis and dermis [94]. The glutathione antioxidant system maintains its capacity until about 45 years of age, but then rapidly declines afterward [95]. This may be one potential mechanism by which age increases the risk to develop PUs [96]. It is reported that non-enzymatic antioxidants are depleted during the early phase of cutaneous wound healing [97]. As consequence, a pro-oxidative shift may happen in cutaneous wounds of elderly people which might attenuate the healing process and promote the development of a PU. Interestingly, antioxidant treatment in aged rats impaired wound healing in the early phase of healing but improved it in the later phases [98]. Thus, oxidative processes may be important in the early phase of wound healing. Patients who are metabolically compromised have a higher risk to develop PUs [99]. A normal functioning metabolism is important to provide the energy and protein substrates that support physiological processes including the immune system. It also provides the antioxidant defense system with diet-derived antioxidants, such as vitamins C and E, and micronutrients such as copper, zinc, and manganese that are required for the activity of superoxide dismutase enzymes [100,101,102].

## 3. Brain Injury and Pressure Ulcers

Traumatic and non-traumatic brain injuries result in patients that are bedridden depending upon the severity of the injury and the related functional impairment. Both in acute and chronic care settings, these patients have been seen to develop PUs [8]. PUs increase the length of hospital stay, extend nursing care, and raise the total treatment cost; they also affect morbidity and mortality, especially in the young [103]. It is reported that immobility is an independent risk factor for PU development besides nutrition, age, gender, associated systemic injury, surgical intervention, and enteral feeding [75]. The role and mechanism of ROS and oxidative stress are likely similar to that discussed for SCI-induced PU formation, although there are no reported studies on the role of ROS and oxidative stress in the context of brain injury. The tools and scales for predicting and reporting the development of PUs are very important in terms of their specificity and sensitivity. The scale of PU prediction after TBI during the acute care setting is very important to provide the proper care for affected patients. According to the GCS score in a different population of mild to severe TBI patients, more patients develop PUs with severe TBI [9] as compared to mild to moderate TBI patients. A study related to Norton and Braden scale estimation/prediction sensitivity and specificity of patients developing PUs suggested that the Braden Scale compared well with the Norton Scale regarding sensitivity, while the specificity was greater for the Norton than for the Braden Scale (64% versus 36%, respectively). This difference is critical to provide the proper care for these patients [104] and assess the overall role of injury in the development of PUs.

Montalcini et al. (2015) studied the brain injury population meeting the minimal conscious state (MCS) criteria and they found that low albumin concentration (<3.1 g/dL) predicted PU formation and mortality. Besides, a low level of hemoglobin was also significantly associated with PU formation in these patients [105]. Dhandapani et al. (2014) found that 16% of TBI patients developed PUs within 21 days of admission despite taking all the measures for PU prevention [10]. TBI-related delayed enteral feeding, >10% decrease in hemoglobin and albumin had a significant impact on PU development with severe TBI. They also reported an association of PUs with recovery status at three months and mortality at 21 days. A study also suggested that patient’s history about their formal life, if it involved planning daily care according to health professionals, had an encouraging effect on PU prevention [106]. A study of TBI patients in long-term palliative care centers (2013–2016) suggested no difference between TBI patients in at-home care vs. rehabilitation centers and intensive care units, and death in respect to mobilization and PUs. PU formation significantly increased the length of stay in TBI patients and had higher GCS scores [107]. Overall, it is not only intrinsic metabolic and physiological changes that are responsible for the formation of PUs after brain injury, but also external factors such as acute primary care and the patient’s history.

## 4. Overview of Clinical Trials

Many clinical trials involved in treating or preventing PUs in SCI and TBI patients target one or more risk factors that benefit the development of chronic PU, such as immobility, a poor health condition of the skin, and/or poor nutrition (Table 1 and Table 2). Direct or indirect treatment of these risk factors might lead to a rescue of the redox balance in the affected skin tissue and recovery from the PU. One strategy is to improve the patients’ and health care workers’ behavior in skincare, and relocation of the patient by educational interviews or apps, telemedicine, or apps that display the pressure on the skin during the patient is in the wheelchair (NCT01885962, NCT01999816, NCT02894437, NCT00763282, NCT00624806, NCT03469141, NCT02800915, NCT04266808, NCT02876666, NCT04309864). As consequence, the risk of bacterial infections, ischemia-reperfusion injury, and inflammation of the skin might be minimized so that the oxidative stress in the skin tissue is attenuated. An optional approach is to treat the symptoms of the chronic PU such as bacterial infections and/or ischemia-reperfusion injury with antibacterial and/or circulation-stimulating substances (NCT01433159, NCT02001558, NCT01500174, NCT02584426). A third strategy to reduce the risk of developing PUs by mechanically changing the pressure of immobile patients over time so that no ischemia-reperfusion injury occurs through mechanical pressure. This can be done by special beds, wheelchairs, gels, bandages, or pulsatile lavage (NCT03317288, NCT03220451, NCT04165395, NCT01943201, NCT03048357, NCT01834417, NCT00047619). Interestingly, there was only one clinical trial that tested a potential beneficial effect of bone marrow mononuclear cells on the wound healing of PUs (NCT01572376, [108]). This is surprising, since it was reported that these cells may attenuate several symptoms that might lead to a PU such as ischemia-reperfusion injury, inflammation, and low antioxidants levels by modulating inflammatory, antioxidant, and apoptotic related molecules [109].

However, to the best of our knowledge, not one single clinical trial is testing an antioxidant treatment on PU treatment in the context of SCI or TBI. This is surprising, since clinical trials that include antioxidants into the diet of patients with PUs observed an improved recovery of the PUs (NCT01107197, [110]; NCT00487097, [111]). In one study with malnourished patients with PUs, a diet supplemented with arginine, zinc, and antioxidants improved PU healing [110]. In the second study, adult patients with PUs of grade II or higher received a diet that is enriched with fish oil. This fish oil supplemented diet improved the wound healing of the PUs and decreased the serum concentration of the inflammatory marker C-reactive protein [111]. Thus, we would like to point out that antioxidant treatment in the form of diet and in combination with a gel that directly affects the PU would be a promising approach to improve the lives of SCI and TBI patients that suffer from PUs.

**Table 1:** Clinical trials for pressure ulcers in the spinal cord injury (SCI) and traumatic brain injury (TBI).

NCT # (Start–End)	Study Title (Phase)	# Enrolled/Completed (M/F)	Recruitment Status	Condition (SCI/TBI)	Treatment	Dose Frequency/Repetition/Duration	Country Co-Sponsor	Results	References
NCT01999816 (2008–2015)	Pressure ulcer prevention study in SCI (Phase 3)	170/NP	Completed	SCI	Pressure Ulcer Prevention Program (PUPP)	One-time home intervention	**USA** University of Southern California	Rates of medically serious pressure injuries were not significantly different across treatment groups.	[112,113,114,115,116,117]
NCT01500174 (2007–2011)	Ultraviolet-C effectiveness in the management of pressure ulcers in people with spinal cord injury (NA)	43/NP	Completed	SCI	Ultraviolet-C therapy	UV-C-radiation 3-time/week until Ulcer close Patient discharge Study period ends	**Canada** Toronto Rehabilitation Institute	In stage 2 buttock ulcers, UVC significantly reduces the % area relative to baseline, but not in stage 3 or 4 ulcers.	[118,119]
NCT01572376 (2007–2010)	Autologous bone marrow stem cells in pressure ulcer treatment (Phases 1 and 2)	30/22 (19/3)	Completed	SCI	Ulcers treated with bone marrow mononuclear cells (BMSCs)	One-time procedure	**Spain** Hospital Universitario Central de Asturias	Patients with (BMSC) treatment reduced 50% stay time in the hospital and also reduced the 75% of daily care requirement in comparison to the patients with conventional surgery.	[108]
NCT00763282 (2008–2014)	Self-management to prevent ulcers in veterans with SCI (spinal cord injury) (NA)	143 (129/4) /78	Completed	SCI	Telephone-based individual MI counseling and SM skills group	8 coordinator-initiated calls over	**USA** US-DVA	No significant increases in skin behaviors between motivational interviewing (MI)/self-management (SM) and control group. High rates of skin worsening (51.7%) were observed in both groups.	[120]
NCT00624806 (2008–2015)	Developing a home telehealth program to manage pressure ulcers in SCI/D (NA)	18 (M)/18 (M)	Completed	SCI	Daily or weekly (depending on treatment group) telephone calls to remind patients how they should prevent ulcers	Daily (56 calls) Weekly (8 calls)	**USA** US-DVA	The daily group had slightly more days of data than the weekly group. Both groups showed a similar number of days with triggers. More participants in the weekly call group experienced equipment issues, skin moisture issues, existing PU care, depression, and ongoing problems affecting self-management than in the daily call group.	[121]
NCT00101361 (2005–2013)	Oxandrolone to heal pressure ulcers (Phase 3)	212 (201/2) /212 (201/2)	Terminated	SCI	Oxandrolone	25 mg/day for 24-week	**USA** US-DVA	Oxandrolone had no significant benefit over placebo for healing.	[122]
NCT00047619 (2001–2008)	Enhancement of pressure healing with pulsatile lavage (Phase 2)	28 (M)/28 (M)	Completed	SCI	Pulsatile lavage treatment	Once-daily for 3 weeks	**USA** US-DVA	A trend of improvement of pulsatile lavage therapy in terms of reducing ulcer length and depth in comparison to the Control group.	[123]
NCT00105859 (2005–2010)	Preventing pressure ulcers in veterans with spinal cord injury (SCI) (Phase—NA)	278/NP	Terminated (recruitment issues)	SCI	Cognitive behavioral intervention	NP	**USA** US-DVA	NP	[124,125,126]
NCT02800915 (2017–2018)	Telemedicine makes the patient stay in hospital at home (NA)	56/NP	Completed	SCI	Interdisciplinary outpatient follow-up via telemedicine	For 12-month or until the pressure ulcer heals	**Norway** Sunnaas Rehabilitation Hospital	NP	[127]

US-DVA—United States Department of Veterans Affairs; NP—Not posted; NA—Not applicable.

**Table 2:** Incomplete or Complete Clinical trials for PU/skin wound in the SCI and TBI.

NCT # (Start–End)	Study Title (Phase)	# Enrolled/Completed (M/F)	Recruitment Status	Condition (SCI/TBI)	Treatment	Dose Frequency/repetition/duration	Country Co-Sponsor	Results	References
NCT01433159 (2011–2014)	Comparison of HP011-101 to standard care for stage I–II pressure ulcers in subjects with spinal cord injury (Phase 2)	19 (M)/16 (M)	Terminated (business decision)	SCI	HP011-101 (Xenaderm Ointment)	Topical ointment applied daily (twice for 14 days)	**USA** Healthpoint	HP011-101 (Xenaderm Ointment) and Standard Care both showed an improved change in scores of the pressure ulcer scale for healing (PUSH)	NP
NCT02001558 (2013–2017)	Pressure ulcer healing with Microcyn (Phase 4)	65 (55/10)/43	Completed	SCI	Microcyn	Topical Microcyn sprayed on wound daily (twice for 24 weeks)	**USA** University of Alabama	Both microcyn and sterile saline (control) reduced ulcer size by more than half of baseline. The PUSH score improved slightly in both treatment groups.	NP
NCT01885962 (2012–2013)	Development and feasibility of an internet intervention for adults with spinal cord injury to prevent pressure ulcers (Phase—NA)	19/NP	Completed	SCI	iSHIFTup: Internet skin health intervention for targeted ulcer prevention	NA	**USA** University of Virginia	NP	NP
NCT03317288 (2017–2019)	Alternating pressure overlay on weight-bearing tissue tolerance in people with spinal cord injury (Phase—NA)	15/NP	Completed	SCI	Device: Dabir Air overlay	NP	**USA** University of Illinois	NP	NP
NCT02584426 (2017–2018)	Subcutaneous injection and ultrasonic dispersion of Cefazolin into chronic pelvic region pressure ulcers in persons with spinal cord injury (Phase—NA)	20/NP	Unknown	SCI	Phonophoresis via ultrasonic distribution of Cefazolin	1 Hypodermic antibiotic injection and Phonophoresis	**USA** James J. Peters Veterans Affairs Medical Center	NP	NP
NCT03220451 (2017–2020)	Use of adhesive elastic taping for the therapy of medium/severe pressure ulcers in spinal cord injured patients (Phase—NA)	24/NP	Recruiting	SCI	Five-layer foam dressing on sacrum and installation of Heelmedix boot alternately from one foot to the other within 48 h after spinal surgery	NP	**Canada** Centre Integre Universitaire de Sante et Services Sociaux du Nord de l’ile de Montreal	NP	NP
NCT02894437 (2016–2018)	A qualitative study of the preventive organization of the pelvic bedsores injured spinal cord (QUALIPREPS) (NA)	45/NP	Unknown	SCI	Conceptual framework for work established to prevent pelvic pressure ulcers	NP	**France** Nantes University Hospital	NP	NP
NCT03469141 (2018–2021)	Interactive telehealth for pressure ulcer prevention after SCI (NA)	100/NP	Recruiting	SCI	Biofeedback via smartphone app	Run biofeedback scan daily for 4 weeks	**USA** Rancho Research Institute, Inc	NP	NP
NCT01943201 (2010–2012)	Low friction bedsheet (NA)	20/NP	Completed	SCI	Low-friction bed sheet	Daily for 5 nights	**Switzerland** Swiss Paraplegic Centre Nottwil	NP	NP
NCT03048357 (2016–2017)	Effectiveness of freedom bed compared to manual turning in the prevention of pressure injuries in persons with limited mobility due to traumatic brain injury and/or spinal cord injury (NA)	8/NP	Unknown	TBI and/or SCI	Freedom bed	Daily for 6 months	**USA** Northeast Center for Rehabilitation and Brain Injury	NP	NP
NCT04402398 (2019–2020)	Psychometric properties of a mobile application (NA)	59/NP	Completed	SCI	imitoMeasure (smartphone app that measures wound size)	NP	**France** University Hospital, Montpellier	NP	NP
NCT04266808 (2020–2022)	Interactive telehealth for wheelchair users (NA)	50/NP	Not yet recruiting	SCI	Interactive telehealth monitoring and biofeedback system on phone application and wheelchair	Used for one year	**USA** Rancho Research Institute, Inc.	NP	NP
NCT02876666 (2017–2017)	Spinal cord injury virtual coach RCT (NA)	40/NP	Completed	SCI	SCI virtual coach interaction	Once-daily for 2 months	**USA** Boston University	NP	NP
NCT01834417 (2013–2020)	Preliminary study leading to prevention of pressure ulcers by the use of an on-board device: Ergonomic assessment of wheelchair-seat pressures in spinal cord injured (SCI) patients (PRESDIE) (NA)	90/NP	Completed	SCI	On-board device (on wheelchair): TexiMat	Use for 4 weeks	**France** Nantes University Hospital	NP	NP
NCT02412046 (2015–2018)	Quantification of the pressure threshold related to tissue injury in bed-ridden paraplegics (NA)	21/NP	Terminated (Departure of the Ph.D. in charge of the study)	SCI	Muscle biopsy after lying on air mattress	One-time procedure	**France** University Hospital, Montpellier	NP	NP
NCT03114345 (2017–2020)	Correlation between pressure differences and micro-vascularization changes in bedridden paraplegic patient (NA)	4/NP	Terminated (Departure of the Ph.D. in charge of the study)	SCI	XSensor	XSensor by bed for 1 h and O2C applied for 1 min; one-time procedure	**France** University Hospital, Montpellier	NP	NP
NCT04309864 (2020–2024)	CMAP refinement for pressure injury prevention (NA)	46/NP	Recruiting	SCI	Comprehensive Mobile Assessment of Pressure (CMAP mobile app)	In-hospital: Use during initial rehabilitation In-home: Use for 2 weeks during daily routine	**USA** VA Office of Research and Development	NP	NP

US-DVA—United States Department of Veterans Affairs; NP—not posted; NA—not applicable.

## 5. Conclusions

In conclusion, the accumulation of ROS in the skin tissue and the resulting oxidative stress is a key mechanism in the formation and impaired healing of PUs. While oxidation seems to have a beneficial effect in the early phase of wound healing, in later phases persistent oxidation causes additional damage to the skin tissue and increases the risk that patients develop a chronic PU. The majority of clinical trials are focusing on the education of the patients, family members, and health care workers to improve the daily care of the skin of SCI and brain injury patients. However, we believe that it is of similar importance to study the effect of antioxidants on PUs in different phases of wound healing in these patients. The use of antioxidants may be incorporated into the daily care of SCI and brain injury patients.

## References

[1] Kruger E.A., Pires M., Ngann Y., Sterling M., Rubayi S. (2013). Comprehensive Management of Pressure Ulcers in Spinal Cord Injury: Current Concepts and Future Trends. J. Spinal Cord Med..

[2] Brem H., Maggi J., Nierman D., Rolnitzky L., Bell D., Rennert R., Golinko M., Yan A., Lyder C., Vladeck B. (2010). High Cost of Stage IV Pressure Ulcers. Am. J. Surg..

[3] Grigorian A., Sugimoto M., Joe V., Schubl S., Lekawa M., Dolich M., Kuncir E., Barrios C., Nahmias J. (2018). Pressure Ulcer in Trauma Patients: A Higher Spinal Cord Injury Level Leads to Higher Risk. J. Am. Coll. Clin. Wound Spec..

[4] Hughes R.G. (2008). Patient Safety and Quality: An Evidence-Based Handbook for Nurses.

[5] Baumgarten M., Margolis D., Doorn C.V., Gruber-Baldini A.L., Hebel J.R., Zimmerman S., Magaziner J. (2004). Black/White Differences in Pressure Ulcer Incidence in Nursing Home Residents. J. Am. Geriatr. Soc..

[6] Ham H.W., Schoonhoven L., Schuurmans M.J., Leenen L.P.H. (2017). Pressure Ulcer Development in Trauma Patients with Suspected Spinal Injury; the Influence of Risk Factors Present in the Emergency Department. Int. Emerg. Nurs..

[7] Garber S.L., Rintala D.H., Hart K.A., Fuhrer M.J. (2000). Pressure Ulcer Risk in Spinal Cord Injury: Predictors of Ulcer Status over 3 Years. Arch. Phys. Med. Rehabil..

[8] Osis S.L., Diccini S. (2020). Incidence and Risk Factors Associated with Pressure Injury in Patients with Traumatic Brain Injury. Int. J. Nurs. Pract..

[9] Teasdale G., Jennett B. (1974). Assessment of Coma and Impaired Consciousness. A Practical Scale. Lancet.

[10] Dhandapani M., Dhandapani S., Agarwal M., Mahapatra A.K. (2014). Pressure Ulcer in Patients with Severe Traumatic Brain Injury: Significant Factors and Association with Neurological Outcome. J. Clin. Nurs..

[11] Paker N., Soy D., Kesiktaş N., Nur Bardak A., Erbil M., Ersoy S., Ylmaz H. (2006). Reasons for Rehospitalization in Patients with Spinal Cord Injury: 5 Years’ Experience. Int. J. Rehabil. Res..

[12] Kottner J., Cuddigan J., Carville K., Balzer K., Berlowitz D., Law S., Litchford M., Mitchell P., Moore Z., Pittman J. (2019). Prevention and Treatment of Pressure Ulcers/Injuries: The Protocol for the Second Update of the International Clinical Practice Guideline 2019. J. Tissue Viability.

[13] Coleman S., Nixon J., Keen J., Wilson L., McGinnis E., Dealey C., Stubbs N., Farrin A., Dowding D., Schols J.M. (2014). A New Pressure Ulcer Conceptual Framework. J. Adv. Nurs..

[14] Bouten C.V., Oomens C.W., Baaijens F.P., Bader D.L. (2003). The Etiology of Pressure Ulcers: Skin Deep or Muscle Bound?. Arch. Phys. Med. Rehabil..

[15] Gawlitta D., Oomens C.W.J., Bader D.L., Baaijens F.P.T., Bouten C.V.C. (2007). Temporal Differences in the Influence of Ischemic Factors and Deformation on the Metabolism of Engineered Skeletal Muscle. J. Appl. Physiol..

[16] Gefen A., van Nierop B., Bader D.L., Oomens C.W. (2008). Strain-Time Cell-Death Threshold for Skeletal Muscle in a Tissue-Engineered Model System for Deep Tissue Injury. J. Biomech..

[17] Stekelenburg A., Strijkers G.J., Parusel H., Bader D.L., Nicolay K., Oomens C.W. (2007). Role of Ischemia and Deformation in the Onset of Compression-Induced Deep Tissue Injury: MRI-Based Studies in a Rat Model. J. Appl. Physiol..

[18] Bader D.L., Barnhill R.L., Ryan T.J. (1986). Effect of Externally Applied Skin Surface Forces on Tissue Vasculature. Arch. Phys. Med. Rehabil..

[19] Dinsdale S.M. (1974). Decubitus Ulcers: Role of Pressure and Friction in Causation. Arch. Phys. Med. Rehabil..

[20] Kosiak M. (1961). Etiology of Decubitus Ulcers. Arch. Phys. Med. Rehabil..

[21] Peirce S.M., Skalak T.C., Rodeheaver G.T. (2000). Ischemia-Reperfusion Injury in Chronic Pressure Ulcer Formation: A Skin Model in the Rat. Wound Repair Regen..

[22] Tsuji S., Ichioka S., Sekiya N., Nakatsuka T. (2005). Analysis of Ischemia-Reperfusion Injury in a Microcirculatory Model of Pressure Ulcers. Wound Repair Regen..

[23] Miller G.E., Seale J. (1981). Lymphatic Clearance during Compressive Loading. Lymphology.

[24] Reddy N.P., Cochran G.V.B., Krouskop T.A. (1981). Interstitial Fluid Flow as a Factor in Decubitus Ulcer Formation. J. Biomech..

[25] Barton A.A., Barton M. (1973). The Medical Management of Pressure Sores. Queens Nurs. J..

[26] Gutteridge J.M.C., Halliwell B. (2018). Mini-Review: Oxidative Stress, Redox Stress or Redox Success?. Biochem. Biophys. Res. Commun..

[27] Halliwell B. (1993). The Chemistry of Free Radicals. Toxicol. Ind. Health.

[28] Dröge W. (2002). Free Radicals in the Physiological Control of Cell Function. Physiol. Rev..

[29] Halliwell B. (1987). Oxidants and Human Disease: Some New Concepts. FASEB J..

[30] Jones S.A., Wood J.D., Coffey M.J., Jones O.T.G. (1994). The Functional Expression of P47-Phox and P67-Phox May Contribute to the Generation of Superoxide by an NADPH Oxidase-like System in Human Fibroblasts. FEBS Lett..

[31] Lo Y.Y.C., Cruz T.F. (1995). Involvement of Reactive Oxygen Species in Cytokine and Growth Factor Induction of C-Fos Expression in Chondrocytes. J. Biol. Chem..

[32] Meier B., Radeke H.H., Selle S., Younes M., Sies H., Resch K., Habermehl G.G. (1989). Human Fibroblasts Release Reactive Oxygen Species in Response to Interleukin-1 or Tumour Necrosis Factor-Alpha. Biochem. J..

[33] Suzuki Y.J., Ford G.D. (1999). Redox Regulation of Signal Transduction in Cardiac and Smooth Muscle. J. Mol. Cell. Cardiol..

[34] Zweier J.L., Broderick R., Kuppusamy P., Thompson-Gorman S., Lutty G.A. (1994). Determination of the Mechanism of Free Radical Generation in Human Aortic Endothelial Cells Exposed to Anoxia and Reoxygenation. J. Biol. Chem..

[35] Babior B.M. (2000). Phagocytes and Oxidative Stress. Am. J. Med..

[36] McCord J.M. (1985). Oxygen-Derived Free Radicals in Postischemic Tissue Injury. N. Engl. J. Med..

[37] D’Autréaux B., Toledano M.B. (2007). ROS as Signalling Molecules: Mechanisms That Generate Specificity in ROS Homeostasis. Nat. Rev. Mol. Cell Biol..

[38] Kehrer J.P. (2000). The Haber–Weiss Reaction and Mechanisms of Toxicity. Toxicology.

[39] Chevion M. (1988). A Site-Specific Mechanism for Free Radical Induced Biological Damage: The Essential Role of Redox-Active Transition Metals. Free Radic. Biol. Med..

[40] Thornberry N.A. (1998). Caspases: Enemies Within. Science.

[41] Halliwell B., Dizdaroglu M. (1992). The Measurement of Oxidative Damage to DNA by HPLC and GC/MS Techniques. Free Radic. Res. Commun..

[42] Drew B., Leeuwenburgh C. (2002). Aging and the Role of Reactive Nitrogen Species. Ann. N. Y. Acad. Sci..

[43] Stamler J., Singel D., Loscalzo J. (1992). Biochemistry of Nitric Oxide and Its Redox-Activated Forms. Science.

[44] Snyder S.H. (1995). No Endothelial NO. Nature.

[45] Wink D.A., Miranda K.M., Espey M.G., Pluta R.M., Hewett S.J., Colton C., Vitek M., Feelisch M., Grisham M.B. (2001). Mechanisms of the Antioxidant Effects of Nitric Oxide. Antioxid. Redox Signal..

[46] Hille R., Hall J., Basu P. (2014). The Mononuclear Molybdenum Enzymes. Chem. Rev..

[47] Chance B., Sies H., Boveris A. (1979). Hydroperoxide Metabolism in Mammalian Organs. Physiol. Rev..

[48] Granger D.N. (1988). Role of Xanthine Oxidase and Granulocytes in Ischemia-Reperfusion Injury. Am. J. Physiol. Heart Circ. Physiol..

[49] Rees R., Smith D., Li T.D., Cashmer B., Garner W., Punch J., Smith D.J. (1994). The Role of Xanthine Oxidase and Xanthine Dehydrogenase in Skin Ischemia. J. Surg. Res..

[50] Shindo Y., Witt E., Han D., Epstein W., Packer L. (1994). Enzymic and Non-Enzymic Antioxidants in Epidermis and Dermis of Human Skin. J. Investig. Dermatol..

[51] Dreher F., Maibach H. (2001). Protective Effects of Topical Antioxidants in Humans. Oxidants and Antioxidants in Cutaneous Biology.

[52] Nabi Z., Tavakkol A., Dobke M., Polefka T.G. (2001). Bioconversion of Vitamin E Acetate in Human Skin. Oxidants and Antioxidants in Cutaneous Biology.

[53] Haslett C. (1992). Resolution of Acute Inflammation and the Role of Apoptosis in the Tissue Fate of Granulocytes. Clin. Sci..

[54] Hourmant M., Vasse N., le Mauff B., Soulillou J.P. (1997). The Role of Adhesion Molecules in Ischaemia-Reperfusion Injury of Renal Transplants. Nephrol. Dial. Transplant..

[55] Perry M.A., Granger D.N. (1991). Role of CD11/CD18 in Shear Rate-Dependent Leukocyte-Endothelial Cell Interactions in Cat Mesenteric Venules. J. Clin. Investig..

[56] Suzuki M., Asako H., Kubes P., Jennings S., Grisham M.B., Granger D.N. (1991). Neutrophil-Derived Oxidants Promote Leukocyte Adherence in Postcapillary Venules. Microvasc. Res..

[57] Salvemini D., Cuzzocrea S. (2002). Superoxide, Superoxide Dismutase and Ischemic Injury. Curr. Opin. Investig. Drugs.

[58] Mittal M., Siddiqui M.R., Tran K., Reddy S.P., Malik A.B. (2014). Reactive Oxygen Species in Inflammation and Tissue Injury. Antioxid. Redox Signal..

[59] Goode H.F., Webster N.R., Howdle P.D., Walker B.E. (1994). Nitric Oxide Production by Human Peripheral Blood Polymorphonuclear Leucocytes. Clin. Sci..

[60] Su Z. (1998). Peroxynitrite Is Not a Major Mediator of Endothelial Cell Injury by Activated Neutrophils in Vitro. Cardiovasc. Res..

[61] Lo S.K., Janakidevi K., Lai L., Malik A.B. (1993). Hydrogen Peroxide-Induced Increase in Endothelial Adhesiveness Is Dependent on ICAM-1 Activation. Am. J. Physiol. Lung Cell. Mol. Physiol..

[62] Patel K.D., Zimmerman G.A., Prescott S.M., McEver R.P., McIntyre T.M. (1991). Oxygen Radicals Induce Human Endothelial Cells to Express GMP-140 and Bind Neutrophils. J. Cell Biol..

[63] Bogdan C. (2001). Nitric Oxide and the Immune Response. Nat. Immunol..

[64] Hampton M.B., Kettle A.J., Winterbourn C.C. (1998). Inside the Neutrophil Phagosome: Oxidants, Myeloperoxidase, and Bacterial Killing. Blood.

[65] Shingu M., Nobunaga M. (1984). Chemotactic Activity Generated in Human Serum from the Fifth Component of Complement by Hydrogen Peroxide. Am. J. Pathol..

[66] Sisley A.C., Desai T., Harig J.M., Gewertz B.L. (1994). Neutrophil Depletion Attenuates Human Intestinal Reperfusion Injury. J. Surg. Res..

[67] Pisoschi A.M., Pop A. (2015). The Role of Antioxidants in the Chemistry of Oxidative Stress: A Review. Eur. J. Med. Chem..

[68] Marzella L., Jesudass R.R., Manson P.N., Myers R.A.M., Bulkley G.B. (1988). Functional and Structural Evaluation of the Vasculature of Skin Flaps after Ischemia and Reperfusion. Plast. Reconstr. Surg..

[69] Granger D.N., Kvietys P.R. (2015). Reperfusion Injury and Reactive Oxygen Species: The Evolution of a Concept. Redox Biol..

[70] Strålin P., Marklund S.L. (1994). Effects of Oxidative Stress on Expression of Extracellular Superoxide Dismutase, CuZn-Superoxide Dismutase and Mn-Superoxide Dismutase in Human Dermal Fibroblasts. Biochem. J..

[71] Sepasi Tehrani H., Moosavi-Movahedi A.A. (2018). Catalase and Its Mysteries. Prog. Biophys. Mol. Biol..

[72] Shi M.M., Kugelman A., Iwamoto T., Tian L., Forman H.J. (1994). Quinone-Induced Oxidative Stress Elevates Glutathione and Induces Gamma-Glutamylcysteine Synthetase Activity in Rat Lung Epithelial L2 Cells. J. Biol. Chem..

[73] Fridovich I., Freeman B. (1986). Antioxidant Defenses in the Lung. Annu. Rev. Physiol..

[74] Speer R.E., Karuppagounder S.S., Basso M., Sleiman S.F., Kumar A., Brand D., Smirnova N., Gazaryan I., Khim S.J., Ratan R.R. (2013). Hypoxia-Inducible Factor Prolyl Hydroxylases as Targets for Neuroprotection by “Antioxidant” Metal Chelators: From Ferroptosis to Stroke. Free Radic. Biol. Med..

[75] Karver C.L., Wade S.L., Cassedy A., Taylor H.G., Stancin T., Yeates K.O., Walz N.C. (2012). Age at Injury and Long-Term Behavior Problems After Traumatic Brain Injury in Young Children. Rehabil. Psychol..

[76] Sies H. (2015). Oxidative Stress: A Concept in Redox Biology and Medicine. Redox Biol..

[77] Marczin N., El-Habashi N., Hoare G.S., Bundy R.E., Yacoub M. (2003). Antioxidants in Myocardial Ischemia–Reperfusion Injury: Therapeutic Potential and Basic Mechanisms. Arch. Biochem. Biophys..

[78] Cohen G.M. (1997). Caspases: The Executioners of Apoptosis. Biochem. J..

[79] Kasahara Y., Iwai K., Yachie A., Ohta K., Konno A., Seki H., Miyawaki T., Taniguchi N. (1997). Involvement of Reactive Oxygen Intermediates in Spontaneous and CD95(Fas/APO-1)—Mediated Apoptosis of Neutrophils. Blood J. Am. Soc. Hematol..

[80] Lundqvist-Gustafsson H., Bengtsson T. (1999). Activation of the Granule Pool of the NADPH Oxidase Accelerates Apoptosis in Human Neutrophils. J. Leukoc. Biol..

[81] Curtin J.F., Donovan M., Cotter T.G. (2002). Regulation and Measurement of Oxidative Stress in Apoptosis. J. Immunol. Methods.

[82] Wolfe J.T., Ross D., Cohen G.M. (1994). A Role for Metals and Free Radicals in the Induction of Apoptosis in Thymocytes. FEBS Lett..

[83] Fernandes R.S., Cotter T.G. (1994). Apoptosis or Necrosis: Intracellular Levels of Glutathione Influence Mode of Cell Death. Biochem. Pharmacol..

[84] Oda T., Sadakata N., Komatsu N., Muramatsu T. (1999). Specific Efflux of Glutathione from the Basolateral Membrane Domain in Polarized MDCK Cells during Ricin-Induced Apoptosis. J. Biochem..

[85] Creagh E.M., Carmody R.J., Cotter T.G. (2000). Heat Shock Protein 70 Inhibits Caspase-Dependent and -Independent Apoptosis in Jurkat T Cells. Exp. Cell Res..

[86] Hampton M.B., Orrenius S. (1997). Dual Regulation of Caspase Activity by Hydrogen Peroxide: Implications for Apoptosis. FEBS Lett..

[87] Leist M., Single B., Naumann H., Fava E., Simon B., Kühnle S., Nicotera P. (1999). Inhibition of Mitochondrial ATP Generation by Nitric Oxide Switches Apoptosis to Necrosis. Exp. Cell Res..

[88] Samali A., Nordgren H., Zhivotovsky B., Peterson E., Orrenius S. (1999). A Comparative Study of Apoptosis and Necrosis in HepG2 Cells: Oxidant-Induced Caspase Inactivation Leads to Necrosis. Biochem. Biophys. Res. Commun..

[89] Amar D., Fleisher M., Pantuck C.B., Shamoon H., Zhang H., Roistacher N., Leung D.H.Y., Ginsburg I., Smiley R.M. (1998). Persistent Alterations of the Autonomic Nervous System after Noncardiac Surgery. Anesthesiology.

[90] Ginsburg I. (1998). Could Synergistic Interactions among Reactive Oxygen Species, Proteinases, Membrane-Perforating Enzymes, Hydrolases, Microbial Hemolysins and Cytokines Be the Main Cause of Tissue Damage in Infectious and Inflammatory Conditions?. Med. Hypotheses.

[91] Ginsburg I. (2002). The Role of Bacteriolysis in the Pathophysiology of Inflammation, Infection and Post-Infectious Sequelae. APMIS.

[92] Durai P.C., Thappa D.M., Kumari R., Malathi M. (2012). Aging in Elderly: Chronological Versus Photoaging. Indian J. Derm..

[93] Kozakiewicz M., Kornatowski M., Krzywińska O., Kędziora-Kornatowska K. (2019). Changes in the Blood Antioxidant Defense of Advanced Age People. Clin. Interv. Aging.

[94] Rhie G., Shin M.H., Seo J.Y., Choi W.W., Cho K.H., Kim K.H., Park K.C., Eun H.C., Chung J.H. (2001). Aging- and Photoaging-Dependent Changes of Enzymic and Nonenzymic Antioxidants in the Epidermis and Dermis of Human Skin In Vivo. J. Investig. Derm..

[95] Jones D.P., Mody V.C., Carlson J.L., Lynn M.J., Sternberg P. (2002). Redox Analysis of Human Plasma Allows Separation of Pro-Oxidant Events of Aging from Decline in Antioxidant Defenses. Free Radic. Biol. Med..

[96] Casimiro C., García-de-Lorenzo A., Usán L. (2002). Prevalence of Decubitus Ulcer and Associated Risk Factors in an Institutionalized Spanish Elderly Population. Nutrition.

[97] Shukla A., Rasik A.M., Patnaik G.K. (1997). Depletion of Reduced Glutathione, Ascorbic Acid, Vitamin E and Antioxidant Defence Enzymes in a Healing Cutaneous Wound. Free Radic. Res..

[98] Khodr B., Khalil Z. (2001). Modulation of Inflammation by Reactive Oxygen Species: Implications for Aging and Tissue Repair. Free Radic. Biol. Med..

[99] Bliss M.R. (1998). Pressure Injuries: Causes and Prevention. Hosp. Med..

[100] Aquilani R., Boschi F., Contardi A., Pistarini C., Achilli M.P., Fizzotti G., Moroni S., Catapano M., Verri M., Pastoris O. (2001). Energy Expenditure and Nutritional Adequacy of Rehabilitation Paraplegics with Asymptomatic Bacteriuria and Pressure Sores. Spinal Cord.

[101] Cruse J.M., Lewis R.E., Dilioglou S., Roe D.L., Wallace W.F., Chen R.S. (2000). Review of Immune Function, Healing of Pressure Ulcers, and Nutritional Status in Patients with Spinal Cord Injury. J. Spinal Cord Med..

[102] Evans P., Halliwell B. (2001). Micronutrients: Oxidant/Antioxidant Status. Br. J. Nutr..

[103] Mervis J.S., Phillips T.J. (2019). Pressure Ulcers: Pathophysiology, Epidemiology, Risk Factors, and Presentation. J. Am. Acad. Dermatol..

[104] Bergstrom N., Demuth P.J., Braden B.J. (1987). A Clinical Trial of the Braden Scale for Predicting Pressure Sore Risk. Nurs. Clin. N. Am..

[105] Montalcini T., Moraca M., Ferro Y., Romeo S., Serra S., Raso M.G., Rossi F., Sannita W.G., Dolce G., Pujia A. (2015). Nutritional Parameters Predicting Pressure Ulcers and Short-Term Mortality in Patients with Minimal Conscious State as a Result of Traumatic and Non-Traumatic Acquired Brain Injury. J. Transl. Med..

[106] Sachs M.B., Wolffbrandt M.M., Poulsen I. (2018). Prevention of Pressure Ulcers in Patients Undergoing Subacute Rehabilitation after Severe Brain Injury: An Observational Study. J. Clin. Nurs..

[107] Kahveci K., Dinçer M., Doger C., Yaricı A.K. (2017). Traumatic Brain Injury and Palliative Care: A Retrospective Analysis of 49 Patients Receiving Palliative Care during 2013–2016 in Turkey. Neural Regen. Res..

[108] Sarasúa J.G., López S.P., Viejo M.Á., Basterrechea M.P., Rodríguez A.F., Gutiérrez A.F., Gala J.G., Menéndez Y.M., Augusto D.E., Arias A.P. (2011). Treatment of Pressure Ulcers with Autologous Bone Marrow Nuclear Cells in Patients with Spinal Cord Injury. J. Spinal Cord Med..

[109] Ornellas F.M., Ornellas D.S., Martini S.V., Castiglione R.C., Ventura G.M., Rocco P.R., Gutfilen B., de Souza S.A., Takiya C.M., Morales M.M. (2017). Bone Marrow–Derived Mononuclear Cell Therapy Accelerates Renal Ischemia-Reperfusion Injury Recovery by Modulating Inflammatory, Antioxidant and Apoptotic Related Molecules. Cell Physiol. Biochem..

[110] Cereda E., Klersy C., Serioli M., Crespi A., D’Andrea F., Perna S. (2015). A Nutritional Formula Enriched With Arginine, Zinc, and Antioxidants for the Healing of Pressure Ulcers A Randomized Trial. Ann. Intern. Med..

[111] Theilla M., Schwartz B., Cohen J., Shapiro H., Anbar R., Singer P. (2012). Impact of a Nutritional Formula Enriched in Fish Oil and Micronutrients on Pressure Ulcers in Critical Care Patients. Am. J. Crit. Care.

[112] Pyatak E.A., Blanche E.I., Garber S.L., Diaz J., Blanchard J., Florindez L., Clark F.A. (2013). Conducting Intervention Research among Underserved Populations: Lessons Learned and Recommendations for Researchers. Arch. Phys. Med. Rehabil..

[113] Blanche E.I., Fogelberg D., Diaz J., Carlson M., Clark F. (2011). Manualization of Occupational Therapy Interventions: Illustrations from the Pressure Ulcer Prevention Research Program. Am. J. Occup. Ther..

[114] Vaishampayan A., Clark F., Carlson M., Blanche E.I. (2011). Individualization of a Manualized Pressure Ulcer Prevention Program: Targeting Risky Life Circumstances Through a Community-Based Intervention for People with Spinal Cord Injury. Adv. Ski. Wound Care.

[115] Fogelberg D., Atkins M., Blanche E.I., Carlson M., Clark F. (2009). Decisions and Dilemmas in Everyday Life: Daily Use of Wheelchairs by Individuals with Spinal Cord Injury and the Impact on Pressure Ulcer Risk. Top. Spinal Cord Inj. Rehabil..

[116] Clark F., Pyatak E.A., Carlson M., Blanche E.I., Vigen C., Hay J., Mallinson T., Blanchard J., Unger J.B., Garber S.L. (2014). Implementing Trials of Complex Interventions in Community Settings: The USC—Rancho Los Amigos Pressure Ulcer Prevention Study (PUPS). Clin. Trials.

[117] Carlson M., Vigen C.L.P., Rubayi S., Blanche E.I., Blanchard J., Atkins M., Bates-Jensen B., Garber S.L., Pyatak E.A., Diaz J. (2019). Lifestyle Intervention for Adults with Spinal Cord Injury: Results of the USC–RLANRC Pressure Ulcer Prevention Study. J. Spinal Cord Med..

[118] Nussbaum E.L., Biemann I., Mustard B. (1994). Comparison of Ultrasound/Ultraviolet-C and Laser for Treatment of Pressure Ulcers in Patients With Spinal Cord Injury. Phys. Ther..

[119] Nussbaum E.L., Flett H., Hitzig S.L., McGillivray C., Leber D., Morris H., Jing F. (2013). Ultraviolet-C Irradiation in the Management of Pressure Ulcers in People With Spinal Cord Injury: A Randomized, Placebo-Controlled Trial. Arch. Phys. Med. Rehabil..

[120] Guihan M., Bombardier C.H., Ehde D.M., Rapacki L.M., Rogers T.J., Bates-Jensen B., Thomas F.P., Parachuri R., Holmes S.A. (2014). Comparing Multicomponent Interventions to Improve Skin Care Behaviors and Prevent Recurrence in Veterans Hospitalized for Severe Pressure Ulcers. Arch. Phys. Med. Rehabil..

[121] Woo C., Guihan M., Frick C., Gill C.M., Ho C.H. (2011). What’s Happening Now! Telehealth Management of Spinal Cord Injury/Disorders. J. Spinal Cord Med..

[122] Bauman W.A., Spungen A.M., Collins J.F., Raisch D.W., Ho C., Deitrick G.A., Nemchausky B.A., Goetz L.L., Park J.S., Schwartz M. (2013). The Effect of Oxandrolone on the Healing of Chronic Pressure Ulcers in Persons With Spinal Cord Injury: A Randomized Trial. Ann. Intern. Med..

[123] Ho C.H., Bensitel T., Wang X., Bogie K.M. (2012). Pulsatile Lavage for the Enhancement of Pressure Ulcer Healing: A Randomized Controlled Trial. Phys. Ther..

[124] Guihan M., Garber S.L., Bombardier C.H., Durazo-Arizu R., Goldstein B., Holmes S.A. (2007). Lessons Learned While Conducting Research on Prevention of Pressure Ulcers in Veterans With Spinal Cord Injury. Arch. Phys. Med. Rehabil..

[125] Guihan M., Garber S.L., Bombardier C.H., Goldstein B., Holmes S.A., Cao L. (2008). Predictors of Pressure Ulcer Recurrence in Veterans With Spinal Cord Injury. J. Spinal Cord Med..

[126] Guihan M., Bombardier C.H. (2012). Potentially Modifiable Risk Factors among Veterans with Spinal Cord Injury Hospitalized for Severe Pressure Ulcers: A Descriptive Study. J. Spinal Cord Med..

[127] Irgens I., Hoff J.M., Sørli H., Haugland H., Stanghelle J.K., Rekand T. (2019). Hospital Based Care at Home; Study Protocol for a Mixed Epidemiological and Randomized Controlled Trial. Trials.

